# Oxidative stress and immune system analysis after cycle ergometer use in critical patients

**DOI:** 10.6061/clinics/2017(03)03

**Published:** 2017-03

**Authors:** Eduardo Eriko Tenório de França, Luana Carneiro Ribeiro, Gabriela Gomes Lamenha, Isabela Kalline Fidelix Magalhães, Thainá de Gomes Figueiredo, Marthley José Correia Costa, Ubiracé Fernando Elihimas Júnior, Bárbara Luana Feitosa, Maria do Amparo Andrade, Marco Aurélio Valois Correia Júnior, Francimar Ferrari Ramos, Célia Maria Machado Barbosa de Castro

**Affiliations:** I Universidade Católica de Pernambuco (UNICAP), Fisioterapia, Recife/PE, Brazil; II Universidade Federal de Pernambuco (UFPE), Fisioterapia, Recife/PE, Brazil; III Universidade de Pernambuco (UPE), Fisioterapia, Recife/PE, Brazil; IV Hospital Agamenom Magalhães (HAM), Fisioterapia, Recife/PE, Brazil; V Hospital Agamenom Magalhães (HAM), UTI Geral, Medicina Intensiva, Recife/PE, Brazil; VI Universidade Federal de Pernambuco (UFPE), Microbiologia, LIKA, Recife/PE, Brazil

**Keywords:** Cytokines, Oxidative stress, Musculoskeletal abnormalities

## Abstract

**OBJECTIVE:**

The passive cycle ergometer aims to prevent hypotrophy and improve muscle strength, with a consequent reduction in hospitalization time in the intensive care unit and functional improvement. However, its effects on oxidative stress and immune system parameters remain unknown. The aim of this study is to analyze the effects of a passive cycle ergometer on the immune system and oxidative stress in critical patients.

**METHODS:**

This paper describes a randomized controlled trial in a sample of 19 patients of both genders who were on mechanical ventilation and hospitalized in the intensive care unit of the Hospital Agamenom Magalhães. The patients were divided into two groups: one group underwent cycle ergometer passive exercise for 30 cycles/min on the lower limbs for 20 minutes; the other group did not undergo any therapeutic intervention during the study and served as the control group. A total of 20 ml of blood was analysed, in which nitric oxide levels and some specific inflammatory cytokines (tumour necrosis factor alpha (TNF-α), interferon gamma (IFN-γ) and interleukins 6 (IL-6) and 10 (IL-10)) were evaluated before and after the study protocol.

**RESULTS:**

Regarding the demographic and clinical variables, the groups were homogeneous in the early phases of the study. The nitric oxide analysis revealed a reduction in nitric oxide variation in stimulated cells (*p*=0.0021) and those stimulated (*p*=0.0076) after passive cycle ergometer use compared to the control group. No differences in the evaluated inflammatory cytokines were observed between the two groups.

**CONCLUSION:**

We can conclude that the passive cycle ergometer promoted reduced levels of nitric oxide, showing beneficial effects on oxidative stress reduction. As assessed by inflammatory cytokines, the treatment was not associated with changes in the immune system. However, further research in a larger population is necessary for more conclusive results.

## INTRODUCTION

Critical patients who require mechanical ventilation (MV) for an extended time period due to underlying disease and adverse effects of drugs undergo an important functional loss. The long immobilization period causes severe osteomyoarticular system dysfunction and is increasingly frequent in these patients 1,2. These irregularities create muscle function losses ranging from a daily decline of 1.3 force to 3 - 10% during an entire week of immobility 3.

Immobilization in bed and increased MV dependence may adversely affect several organs and systems, leading to the following consequences: muscle contractures; functional loss; reduced maximal oxygen uptake (VO_2_ Max); muscle weakness in the intensive care unit (ICU); deep venous thrombosis; pressure ulcers; pneumonia; atelectasis; bone demineralization; and changes in the emotional state such as anxiety, apathy and depression 4.

Muscle weakness, which is common in critical patients, is associated with an inflammatory dysregulation that appears to contribute to myopathy. The mechanism for muscle decay due to immobility has not yet been completely clarified. Two molecular interactions are involved: oxidative stress and selected proinflammatory cytokines. It is believed that this synergy between oxidative stress, inflammatory cytokines and inactivity causes or accelerates muscular atrophy 5,6.

Many studies have been developed with the objective of preventing the deleterious effects of ICU-acquired paresis or to minimize it, as an alternative therapy. Amongst the recommended procedures are passive exercises and/or activities, daily sedation suspension and reduced infusion of drugs such as neuromuscular blockers and corticosteroids, the maintenance of homeostasis of electrolytes and nutritional intake 7.

The effects of physical exercise in critical patients for the prevention of atrophy and muscle strength improvement with a consequent reduction in hospitalization time in intensive care and functional improvement have rapidly expanded in recent years. However, changes in oxidative stress and immune system parameters of these patients are not well defined and require further study, thus justifying the aim of this research of improving our understanding of the effect of isolated passive cycle ergometry use in critical patients. Therefore, this study analyses the oxidative stress and the immune system parameters after passive cycle ergometry use on lower limbs in critical patients.

## MATERIALS AND METHODS

This was a randomized controlled trial, with a sample consisting of 19 MV patients of both genders hospitalized in the ICU of Hospital Agamenom Magalhães (HAM) who met the inclusion criteria. The study protocol was conducted in the ICU of HAM, but the blood analysis was performed in the laboratory of Immunology Keizo Asami (LIKA) at the Universidade Federal de Pernambuco. This study was approved by the Ethics and Research Committee (ERC) of the hospital under CAAE number 04563612.5.0000.5197, and all legal guardians of the patients signed informed consent (TFCC).

The patients who underwent MV presented a good cardiovascular reserve, demonstrated by less than 20% heart rate (HR) variability, systolic blood pressure (SBP) less than 200 mmHg or greater than 90 mmHg, normal electrocardiogram (no evidence of acute myocardial infarction or arrhythmia) and a good respiratory reserve, demonstrated by a peripheral oxygen saturation (SpO_2_) greater than 90% and an inspired fraction of oxygen (FiO_2_) less than 60%, without signs of respiratory distress and a respiratory rate (RR) less than 25 ipm. Other clinical parameters necessary for inclusion in this study were as follows: stable haemoglobin of >7 g/dL, stable platelet count of >20,000 cells/mm^3^, white blood cell count of 4,300 - 10,800 cells/mm^3^, body temperature <38°C and blood glucose levels of 3.5 – 20 mmol/L. Other parameters included an acceptable patient appearance, pain, fatigue, shortness of breath and emotional status; a stable conscious state, no other neurological contraindications, no orthopaedic contraindications, no recent SSG / flap to lower limbs or trunk, medically stable without vasoactive drugs and/or minimal doses, excessive weight able to be safely managed, no attachments that contraindicated mobilisation, safe environment, appropriate staffing and expertise and patient consent.

Patients who presented with hemodynamic instability, the inability to walk without assistance before acute disease in the ICU, under 21 years old, pregnant women, patients with a body mass index (BMI) greater than 35 Kg/m^2^ 2, neuromuscular disease or vascular disease, cerebrovascular accident history, non-consolidated fractures or any osteomyoarticular limitation that precludes cycle ergometry use were excluded from the study.

After being recruited to the study, the participants were evaluated through medical records, demographic information, medical history and diagnosis. Data on neuromuscular blockers, sedatives and vasoactive drugs were also collected.

When this initial review of all patients was completed, they were submitted to blood collection by central venous access, both before and 1 hour after the study protocol finalization. For each patient, a total of 20 ml of blood was collected with vacuum tubes (Vacutainer ®) DIPOTASSIUM EDTA (Juiz de Fora, Minas Gerais, Brazil) to evaluate the oxidative stress and immune system parameters as indicated by the cytokines tumour necrosis factor alpha (TNF-α), interferon gamma (IFN-γ) and interleukins 6 (IL-6) and 10 (IL-10).

Oxidative stress was evaluated with EDTA in monocytes obtained from the peripheral blood. The blood was diluted at a ratio of 1:2:00 pm with sterile PBS culture medium at a room temperature of 22 to 25° C (10 ml+10 ml blood in PBS). A total of 10 ml of histopaque (1077-SIGMA) was added to 20 ml of the suspension, and all contents were centrifuged for 30 minutes at 1,600 rpm (25° C). Soon after, the plasma was aspirated, and the layer formed by the cells was collected (PBMC) and transferred to another test tube. The same amount of aspirated PBS was added and centrifuged for 15 minutes under the same conditions as before. The supernatant was decanted and sedimented in 1 ml of RPMI 1640 medium containing 3% bovine foetal serum and antibiotics (100 U/ml penicillin and 100 µg/ml streptomycin). The suspension calculation was performed in a Neubauer chamber assuming the rate of adding a suspension of cells and trypan blue dye in a 1:10 dilution. This dye was used for cell calculation and for assessing their viability. It was standardized to a concentration of 1×10^6^ cells for each 1 ml of culture medium from the measurements.

### Nitric oxide (NO) production in cultured monocytes treated with *Escherichia coli* lipopolysaccharide (LPS)

In each group, the concentration was adjusted to 1×106 cells in 1 ml of culture medium to each well. The cells were treated at a dose of 10 µg/ml of LPS for 24 hours. The NO evaluation release was performed using the GRIESS method. First, 50 µl of GRIESS reagent (1 g of sulphanilamide, Sigma 9251; 0.1 g of N- (naphthyl) ethylenediamine dihydrochloride, Sigma 5889; 2.5 ml of phosphoric acid PA; and qsp 100 ml of distilled water) was added. Then, the plate was incubated for 10 minutes in the dark. The reading was performed at 540 nm in an ELISA reader (Dynatech MR 5000). The sensitivity test threshold was 1.56 µM.

### Analysis of immune system parameters by quantification of IL-6, IL-10, TNF-α and IFN-γ

Serum levels of IL-6, IL-10, TNF-α and IFN-γ were determined using ELISA commercials kits for IL-6, Il-10, TNF-α and IFN-γ (BioSource ®, Nivelles, Belgium, Europe) according to the manufacturer’ instructions. In this technique, a specific monoclonal antibody is adsorbed to the plate. After the serum sample addition of the mediator to be dosed, during incubation, the molecules of antigens settle to the antibodies adsorbed to the plate. Through washing, any non-fixed materials are eliminated. Next, new antibodies with specificity for an antigenic determinant connected to the plate are added, resulting in the complex Bc-Ag-Ac-enzyme (sandwich technique). A second wash is performed for the removal of unlinked antibodies. Then, a substrate is added that has the property of assuming a different coloration when in contact with the enzyme; this colour is proportional to the amount of mediator present in the sample (antigen). The reading is obtained in a plate reader (Bio-Rad, Tokyo, Japan) at 450 nm and compared to a standard curve obtained with known concentrations of recombinant mediators.

### Study protocol

**Figure 1:**
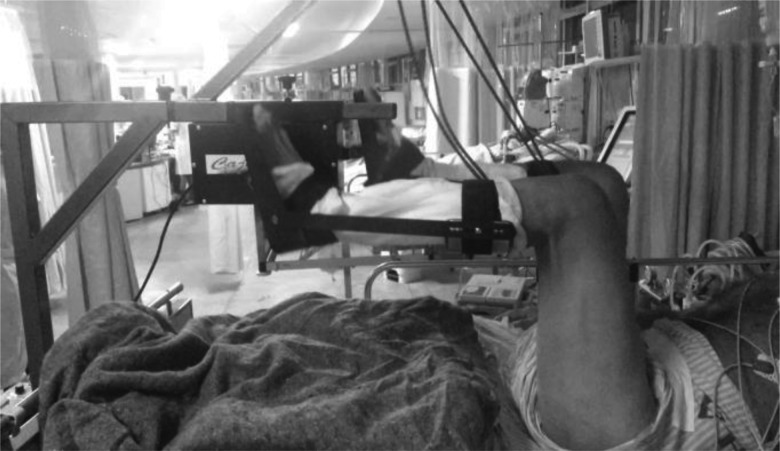
Illustration of passive cycle ergometer application on lower limbs in critical patients under mechanical ventilation.

The study population was divided into two groups: the control group, consisting of 10 patients who were without any type of therapeutic intervention at the moment when they were submitted to the study protocol, and the intervention group, consisting of 9 patients who were submitted to passive cycle ergometry on their lower limbs with the speed adjusted at 30 cycles per minute for 20 minutes, by a cycle ergometer (Flex Motor with sensor; Cajumoro; Bragança Paulista, Sao Paulo, Brazil). Figure 1 demonstrates the protocol in the lower limbs. The participation in one of the two groups in this study was randomly determined by Microsoft Office Excel 2007.

All results and demographic characteristics were assessed using GraphPad Prism 4 software and Microsoft Office Excel 2007. The presentation of the measured variables was performed through tables and figures. The median and the percentile (25-75%) were used to present continuous variables, whereas categorical data were presented using absolute and relative frequencies.

To test the normality assumption of the variables in the study, the Shapiro-Wilk test was used. The comparative analysis between two groups was performed using the Mann-Whitney test, and the Wilcoxon test was used to compare the same group. Fisher's exact test was used to evaluate the differences between the proportions. The relationship between variables was assessed using the Spearman correlation (non-normal distribution). All findings were considered at a 5% significance level.

## RESULTS

**Figure 2:**
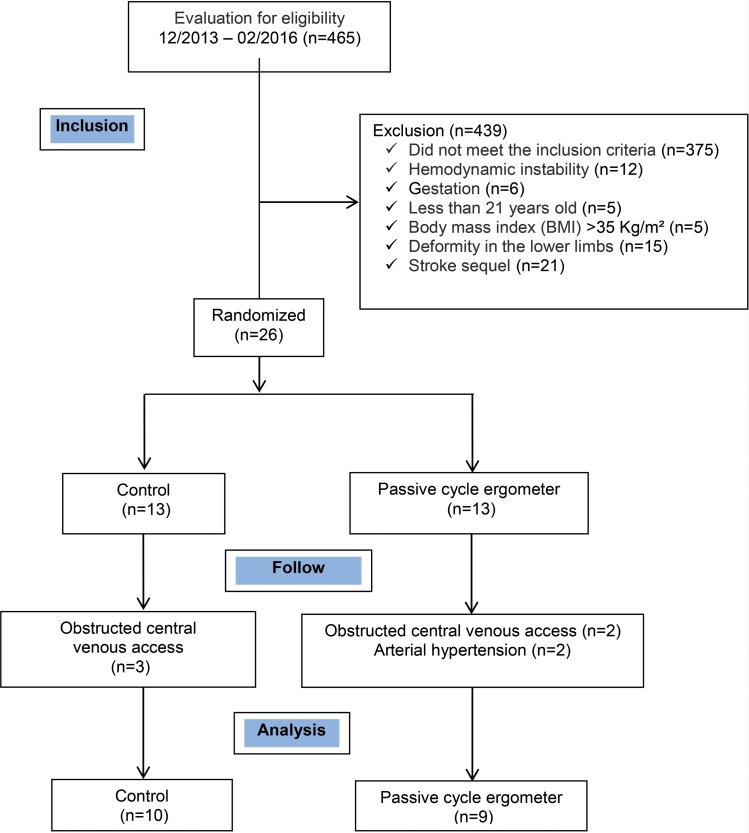
Flowchart of the patients who participated in the study.

During the study period, from December 2013 to February 2016, a total of 465 patients with various diseases were admitted to the general ICU, of whom 439 individuals met the exclusion criteria of the study. Only 26 patients were randomized into two groups; amongst these, only 19 patients finished their analysis, distributed as follows: control group (n=10) and exercise group (n=9) (Figure 2).

Table 1 presents the median and percentile (25 - 75%) of the demographic and clinical variables of each of the two groups: control and cycle ergometer. There were no differences between the two groups regarding age, height, weight, BMI, APACHEII, water balance (BH) in the last 12:00 am, sedation scale of RASS, MV, hospitalization time in ICU, hemoglucotest (HGT), respiratory system compliance (Cst), respiratory system resistance (Rsr), HR, SpO_2_, SBP, diastolic blood pressure (DBP), temperature (T) and ICU mortality, demonstrating the homogeneity between the groups.

**Figure 3a and 3b:**
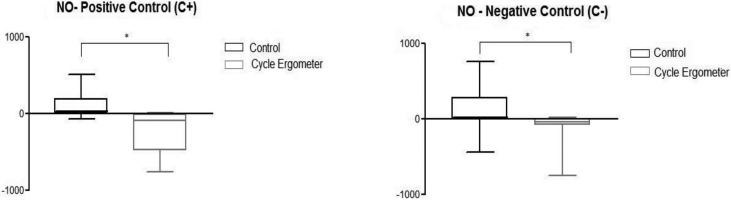
Variation in the nitric oxide (NO) values in stimulated control positive cell (C +) and unstimulated in the two groups studied: control and passive cycle ergometer. * Mann-Whitney Test. Differences between the control group and cycle ergometer 3a. (*p=0.0021) and 3b. (*p=0.0076).

Figures 3a and 3b represent the results obtained from the NO analysis of stimulated monocytes (positive controls) and unstimulated (negative control) collected before and after the study protocol from both groups. These figures revealed a reduction in NO variation in the production of stimulated monocytes (*p*=0.0021) and unstimulated (*p*=0.0076) after passive cycle ergometry use compared to the control group.

Table 2 shows the median and percentile (25 - 75%) values for the cytokines, TNF-α, IFN-γ, IL-6 and IL-10, analysed before and after the study protocol for each group, in which there were no significant changes in cytokines compared to the moments before and after for the two groups.

Correlations were made between cytokines (TNF-α, IL-6, IL-10 and IFN-γ) and NO concentration in the stimulated cells, positive control (C+) and negative control (C-) without any significant difference at *p*>0.05.

## DISCUSSION

There is no evidence in the literature that describes the effects of a passive cycle ergometer on oxidative stress and immune system parameters in critical patients. However, several other benefits of this intervention have been widely observed, particularly regarding muscle mass loss and improved functionality.

During the period that these patients are restricted to bed, muscle fibre transformation to type II occurs, including reduced oxidative capacity, mitochondrial density and blood capillaries. In addition, the cardiovascular performance is reduced due to lower systolic ejection volume and increased HR. Venous stasis occurs due to reduced activity of the muscle pump in the limbs and an increased risk of developing thrombosis. The period of immobility can also contribute to bone demineralization and sodium and body water reduction 8.

In the present study, the values described in Table 1 show that no differences were found in the demographic and clinical characteristics of the patients in both groups, which demonstrates the homogeneity between groups for all evaluated parameters. These partial results of homogeneity between the groups are important because they do not expose any group to a greater risk factor for its clinical condition commitment. Muscle dysfunction in critical patients is a common clinical condition in patients submitted to prolonged MV periods and hospitalization in intensive care. The magnitude of muscle weakness in the ICU is extremely variable and presents an intimate association with permanence time in bed and exposure to risk factors, such as nutritional condition and MV dependence 4,9.

Critical patients are vulnerable to synthesizing oxidative agents and reducing antioxidants. Oxidative stress increases oxidation and has an important role in the pathophysiological process of muscular dysfunction. Reactive oxygen species (ROS) provide lipid peroxidation, leading to toxin release and arachidonic acid derivatives that damage cell membranes, inactivating the receptor membrane enzymes and ionic chemistry response changes. In myocytes, this process of degradation can be modified by numerous intracellular signals observed in vitro, including the activation of nuclear factor kappa-β (NF-k β) 10. Products of cell destruction by ROS produce a positive *feedback*, generating more ROS. Simultaneously, the interaction between ROS with cytokines and other intercellular molecules is associated with muscle degradation.

Figures 3a and 3b show that for both stimulated monocytes (C+) and unstimulated monocytes (C-), there was a reduction in NO values in the cycle ergometer passive group compared to those in the control group. NO values decreased before compared with after the intervention, a phenomenon not observed in the control group.

These findings suggest a potential beneficial effect of passive cycle ergometry in oxidative stress reduction in critical patients; it can be considered a moderate-intensity physical activity for this type of patient, providing a positive change in the redox status of cells and tissues from basal levels, decreasing oxidative damage and increasing resistance to oxidative stress 11-13. In fact, regular moderate exercise results in adjustments in antioxidant capacity, which protects cells against the damaging effects of oxidative stress, preventing subsequent cell damage 14,15.

Our results correlate with the findings of Mercken et al. 16, who assessed the different intensities of exercise and its effects on oxidative stress, immediately after and 4 hours after the cycle ergometer test with 60% workload, in patients with chronic obstructive pulmonary disease (COPD). There was a reduction of the induction of systemic oxidative stress triggered by exercise, particularly after submaximal exercises.

Similar to oxidative stress, some selected cytokines also influence muscle degradation and dysfunction in critical patients. In a model to explain cachexia, Reid and Li 17 suggested that the interaction between ROS and proinflammatory cytokines such as TNF-α are synergistic, perhaps indicative of a pathological positive feedback cycle, which is lower in the regulation of repairing damaged muscle tissue. Thus, it is not only the direct suppression of muscle activity that leads to dysfunction in the presence of TNF-α, but a decrease in repairing and/or an increase of apoptosis that results in muscle weakening of these mediated inflammatory cytokines.

Table 2 shows that there was no significant difference between the values of TNF-α, IFN-γ, IL-6 and IL-10 compared to the moments before and after the cycle ergometer intervention. Comparing the cycle ergometer and control groups demonstrated that the passive cycle ergometer in this study was not enough to promote changes in the immune system after 1 hour of passive exercise. The level of exercise intensity and the kinetics of cytokines may have been responsible for the lack of changes in serum levels of these cytokines in the studied groups.

According to Winkelman et al. 18, in healthy individuals, increased TNF-α varies in response to exercise; it is greater when oxidative stress is present during the exercise. Serum levels of TNF-α also reduce antioxidant levels in some skeletal muscles. Because TNF-α is a proinflammatory cytokine, it stimulates the synthesis of several factors, including adhesion molecules, and ROS.

Another very important cytokine in the inflammatory process is IL-6, which has a wide range of biological activities. It is synthesized by the immune system and skeletal muscle cells, adipocytes, endothelial cells and intestinal epithelium cells 19. Similar to TNF-α, IL-6 is released early in the inflammatory cascade. Unlike TNF-α, IL-6 increases in myosin and appears to have a role in the maintenance of the power supply during the lifetime of the myocyte.

Muscle contraction induces IL-6 production and release to the plasma in large quantities. An increase of up to 100 times the initial value can be found in some studies in humans. The synthesis of IL-6 during exercise is independent of the production of TNF-α. During exercise, even low intensity, IL-6 is also synthesized and put into circulation by the muscle-tendon and fatty tissues 20.

Apart from its proinflammatory properties, high levels of IL-6 act to stimulate the emergence of antiinflammatory cytokines in the plasma, including IL-10 and IL-1R α. IL-6 derived from muscles reduces the production of TNF-α, interrupting the muscle degradation through the destruction of myosin 18.

The abolition of proinflammatory cytokines such as IL-6, particularly the powerful IL-β 1 and TNF-α, can benefit the critically ill. IL-10 is an anti-inflammatory cytokine that is initially identified by its ability to interrupt the production of cytokines by T cells. Investigations have shown that IL-10 inhibits the synthesis of IL-1 β, IL-6, TNF-α, reactive oxygen intermediates and other proinflammatory factors, suppressing various immune responses through individual actions on multiple cell types.

After exercise, high circulating IL-6 levels are followed by increased production of IL-10. Studies suggest that exercise has an antiinflammatory action by inducing IL-10 and IL-6 and inhibiting TNF-α and IL-β 1. The cellular messengers IL-6 and IL-10 are involved in the maintenance of muscle function during stretching and some exercise types. Exercise is used as an inflammation regulator and muscle function 18. The truth is that important clinical implications have been demonstrated with physical exercise and neuromuscular electrical stimulation (NMES) as some of the main prevention factors in the muscle function of critical patients.

Although changes in inflammatory cytokines in a single session using the cycle ergometer were observed, we believe that the implementation of the passive cycle ergometer has beneficial effects on this response, similar to a report by Karavidas et al. 21 who studied the application of six weeks of NMES in the lower limbs of patients with severe heart disease. They observed that NMES could promote a direct effect on endothelial function and the peripheral markers of antiinflammatory activation with reduced levels of TNF-α and IL-6 and improved blood flow in the brachial artery observed by ultrasound with doppler. These cytokines may systemically act as anabolic muscle and enhance stimulus catabolic effects of critical illness and paralysis. NMES and exercise can also activate a bioenergetics pathway that systemically improves the mitochondrial function of skeletal muscles.

In another study evaluating the effects on the immune system, after a total of 20 sessions, Akar Olcay et al. 22 studied patients with COPD under MV whose objective was to investigate the effect of active mobilization and NMES in the weaning process, discharge and inflammatory mediators. A total of 30 patients were divided into three groups with 10 patients in each group: group 1 was submitted to active mobilization of extremities and NMES, group 2 was submitted to NMES, and group 3 only underwent active mobilization of extremities. Significant improvement was observed in peripheral muscle strength, particularly of the lower extremities, in the groups that performed NMES and exercises and NMES exclusively. In addition, a reduction of IL-6 and IL-8 in patients submitted to NMES was observed.

It is too early to draw any firm conclusions from our findings. However, we can conclude that passive cycle ergometry on the lower limbs was enough to reduce NO levels in cells compared to the control group, showing the benefits of passive exercise on oxidative stress reduction in the study population. Regarding the behaviour of the inflammatory cytokines evaluated in this study, we observed that the use of the passive cycle ergometer did not cause changes in the immune system. However, additional research in a larger population is necessary for more conclusive results.

## AUTHOR CONTRIBUTIONS

All of the authors participated in the design, interpretation of studies, data analyses and review of the manuscript. França EE, Ribeiro LC, Lamenha GG, Magalhães IK, Figueiredo TG, Costa MJ, Elihimas-Júnior UF and Barbara Feitosa BL participated in the realization of experiments and laboratory analysis of oxidative stress and inflammatory cytokines. Andrade MA and Castro CM guided the research elaboration. Correia-Júnior MA and Ramos FF wrote the manuscript and performed the statistical analysis.

## Figures and Tables

**Table 1:** Comparison of demographic and clinical variables between the two groups.

Demographic and clinical variables	Control (n=10)	Passive cycle ergometer (n=9)	*p* * value
Age (years)	56.0 (44.0 – 70.5)	77.0 (32.5 – 81.0)	0.35
Height (cm)	164.5 (157.5 -172.0)	160.0 (153.5 – 162.5)	0.09
Weight (Kg)	67.5 (60.0 -80.0)	70.0 (55.0 – 80.0)	0.84
BMI (Kg/cm^2^)	25.1 (23.9 – 26.1)	27.3 (22.1 – 33.1)	0.40
APACHE II	23.0 (19.0 – 27.0)	25.0 (15.5 -29.5)	0.90
WB-12:00 am (ml)	1131.0 (200.5 – 1811.0)	487.0 (343.0 – 1341.0)	0.60
MV T (days)	4.0 (2.5 – 8.0)	5.0 (4.0 – 8.0)	0.11
ICU T (days)	4.0 (2.5 – 8.0)	6.0 (5.0 -8.0)	0.06
HGT	130.5 (92.5 – 176.5)	122.0 (88.0 – 177.5)	0.06
Cst (ml/cm H_2_O)	32.2 (27.1 – 37.1)	27.0 (17.6 – 33.9)	0.15
Rsr (cm H_2_O/L/s)	12.0 (9.2 – 18.0)	10.0 (10.0 – 16.0)	0.66
FC (bpm)	83.0 (61.0 – 99.0)	86.0 (78.5 – 94.5)	0.60
SpO_2_ (%)	98.5 (94.0 – 99.5)	98.0 (97.5 – 99.0)	0.71
SBP (mm Hg)	137.5 (108.5 – 162.5)	142.0 (125.0 – 151.0)	0.71
DBP (mm Hg)	80.0 (62.0 – 93.0)	74.0 (66.0 – 88.5)	0.84
Temperature (^0^C)	36.0 (36.0 - 36.5)	36.5 (36.5 – 37.0)	0.21
ICU mortality	6 (60.0)	7 (77.7)	0.63
Primary reason for admission			
Respiratory problem	4 (40.0)	4 (44.4)	
Cardiac problem	2 (20.0)	2 (22.2)	–
Sepsis/infection	1 (10.0)	2 (22.2)	
Other	3 (30.0)	1 (11.1)	
Comorbid conditions			
Respiratory	2 (20.0)	1 (11.1)	
Cardiac	2 (20.0)	3 (33.3)	
Endocrine	2 (20.0)	1 (11.1)	–
Urinary	1 (10.0)	1 (11.1)	
Chronic renal failure	3 (30.0)	2 (22.2)	
Sepsis/infection	1 (10.0)	1 (11.1)	

Data are the median (25 -75% percentile) before testing.

* Mann-Whitney test and Fisher’s exact test.

Body mass index (BMI); Acute Physiology and Chronic Health Evaluation (APACHE II); water balance in the last 24 hours (WB-24 h); mechanical ventilation time (MV T); intensive care unit time (ICU T); hemoglucotest (HGT); static compliance of the respiratory system (Cst); resistance of the respiratory system (Rsr); heart rate (HR); peripheral oxygen saturation (SpO_2_); systolic blood pressure (SBP); diastolic blood pressure (DBP); temperature and intensive care unit (ICU) mortality.

**Table 2:** Comparison of cytokine measurements before and after for the control and passive cycle ergometer groups.

Cytokines	Control (n=10)		Passive cycle ergometer (n=9)		
	Before	After	Before	After	
**TNF-α**	3.05 (2.83-3.22)	2.91 (2.70-3.30)	3.35 (2.72-3.59)	2.87 (2.74-3.28)	
	*p*=0.625		*p*=0.301		p* value
**IFN-γ**	1.88 (1.87-1.91)	1.88 (1.86-1.91)	1.91 (1.86-1.95)	1.92 (1.87-1.98)	
	*p*=0.152		*p*=0.944		p* value
**IL- 6**	2.87 (2.42-3.86)	2.67(2.33-3.61)	2.37 (2.30-2.96)	2.39 (2.23-2.88)	
	*p*=0.695		*p*=0.820		p* value
**IL- 10**	1.86 (1.83-1.93)	1.85 (1.83-1.93)	1.91 (1.88-1.93)	1.92 (1.91-1.93)	
	*p*=0.998		*p*=0.528		p* value

Proinflammatory cytokines (TNF-α, IFN-γ and IL-6) and antiinflammatory properties (IL-10).

Values written in bold are statistically significant.

Data are the median (25 – 75% percentile) before and after testing.

* Wilcoxon test.

TNF-α, tumour necrosis factor alpha; IFN-γ, interferon gamma; IL-6, interleukin 6; IL-10, interleukin 10.
